# Diagnostic Accuracy of Abdominal wall Ultrasonography and Local Wound Exploration in Predicting the Need for Laparotomy following Stab Wound

**Published:** 2017-01-11

**Authors:** Ali Vafaei, Kamran Heidari, Afshin Saboorizadeh, Amin shams akhtari

**Affiliations:** 1 Department of Emergency Medicine, Loghman Hakim Hospital, Shahid Beheshti University of Medical Sciences, Tehran, Iran.

**Keywords:** Abdominal injuries, wounds, penetrating, wounds, stab, ultrasonography, diagnostic techniques and procedures

## Abstract

**Introduction:**

Screening of patients with anterior abdominal penetrating trauma in need for laparotomy is an important issue in management of these cases. This study aimed to compare the accuracy of abdominal wall ultrasonography (AWU) and local wound exploration (LWE) in this regard.

**Methods:**

This diagnostic accuracy study was conducted on ≥ 18 year-old patients presenting to emergency department with anterior abdominal stab wound and stable hemodynamics, to compare the characteristics of AWU and LWE in screening of patients in need of laparotomy.

**Results:**

50 cases with the mean age of 28.44 ± 7.14 years were included (80% male). Sensitivity, specificity and area under the receiver operating characteristic (ROC) curve of AWU were 70.58 (95% CI: 44.04 – 88.62), 93.33 (95% CI: 76.49 – 98.83), and 81.96 (95% CI: 69.91 – 94.01), respectively. These measures were 88.23 (62.25 – 97.93), 93.33 (76.49 – 98.83), and 90.78 (95% CI: 81.67 – 99.89) for LWE, respectively. The difference in overall accuracy of the two methods was not statistically significant (p = 0.0641).

**Conclusion:**

Based on the findings of the present study, AWU and LWE had the same specificity but different sensitivities in screening of anterior abdominal stab wound patients in need of laparotomy. The overall accuracy of LWE was slightly higher (91.48% versus 85.1%).

## Introduction

Penetrating abdominal trauma is known as one of the relatively frequent causes of emergency department visits. There is a general agreement that patients with penetrating abdominal trauma and hemodynamic instability must immediately be referred to operation room without additional diagnostic measures (1). However, management of hemodynamically stable patients is a controversial issue. At least 25% of anterior abdominal stab wounds are superficial and do not need laparotomy (2). Unnecessary laparotomy can increase the risk of morbidity and mortality of these patients (3). Sanei et al. showed that 82% of stab wound laparotomies, which were done only based on anterior fascial impairment, are negative (4).

 In recent years, most physicians have tried to change their diagnostic approaches from mandatory exploration to selective diagnostic approaches (5-7). Appropriate approach to these patients needs balance between invasiveness and accuracy of diagnostic measures in this regard. Using ultrasonography, deep peritoneal lavage (DPL), serial clinical examination, local wound exploration (LWE), and abdominal computed tomography (CT) scan are suggested by different studies to decrease the rate of unnecessary laparotomies.

LWE is a valid, yet invasive, screening tool for selection of stab wound patients in need of laparotomy (2). However, abdominal wall ultrasonography (AWU) seems to be as a non-invasive, available, bedside, and safe alternative for LWE in this regard (1, 3). Based on the above-mentioned point, this study aimed to assess the diagnostic accuracy of AWU and LWE in detection of patients in need of laparotomy following anterior abdominal stab wound. 

## Methods

***Study design and setting***

This cross sectional study was conducted on patients presenting to emergency departments of Loghman Hakim, Imam Hossein, and Hafte-tir Hospitals, Tehran, Iran, following anterior abdominal stab wound, during March 2013 to March 2015, to compare the diagnostic accuracy of AWU and LWE in prediction of need for laparotomy. The study protocol was approved by ethics committee of Shahid Beheshti University of Medical Sciences and informed consent form was signed by all participants. Authors adhered to all Helsinki recommendations and confidentiality of patients’ information during the study period.

***Participants***

Patients older than 18 years old with anterior abdominal stab wound were included. Hemodynamic instability, presenting peritoneal signs, protrusion of abdominal organs, gastrointestinal bleeding, pregnancy, instrument in situ, presence of abdominal free fluid on focused abdominal ultrasonography for trauma patients (FAST), peritoneal evisceration, multiple wounds, and need for emergent laparotomy were among the exclusion criteria. 

Anterior abdominal wall was defined as the area superior to the inguinal ligaments, medial to the anterior axillary line, and two fingerbreadths inferior to the costal margins. 

***Data gathering***

A predesign check list, consisting of demographic information (age, sex), trauma mechanism, vital signs (blood pressure, heart rate, respiratory rate, and oxygen saturation), findings of AWU and LWE regarding need for laparotomy, as well as final outcome (decision of in charge surgeons regarding performing laparotomy), was used for data gathering. 

Without interfering in the routine approach, eligible patients underwent AWU by a trained senior emergency medicine resident (under supervision of an emergency medicine specialist) at the time of admission to emergency department. The emergency medicine resident was trained and certified by an expert radiologist, performing tract ultrasonography on ten sheep cadaver models under his direct supervision.

After doing AWU, all patients underwent LWE by senior surgery residents and were followed until discharge from hospital. 

Patients that underwent laparotomy based on final decision of in charge surgeon, considering all clinical and imaging findings (serial clinical examination, abdominal CT scan, serial FAST, and etc.) during the period of hospital admission were considered as reference group. 

**Figure 1:**
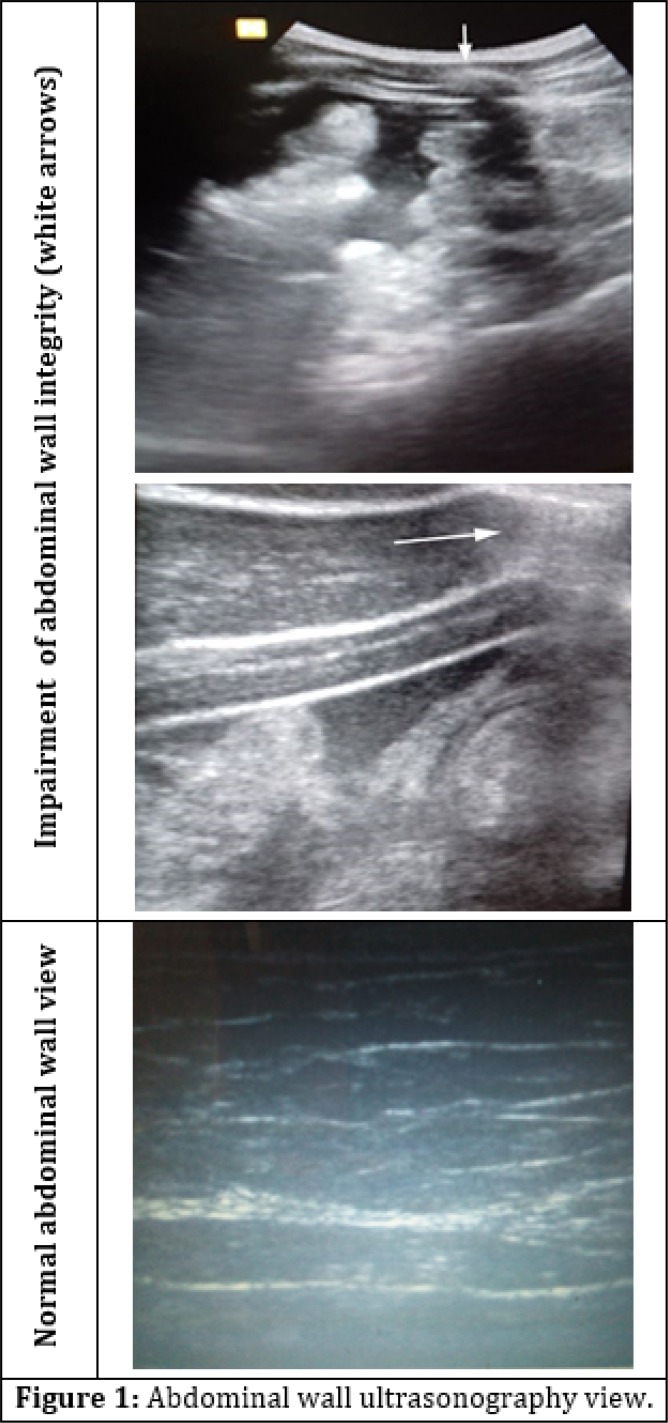
Abdominal wall ultrasonography view.

Samsung HM70A ultrasonography machine with 8 MHZ linear probe was used for ultrasonography of the abdominal wall and its 10 x 10 cm surrounding area (figure 1). 

***Statistical analysis***

Considering 25% prevalence of penetrating abdominal trauma in need for laparotomy (2), d = 0.05, and 95% confidence interval (CI), the minimum required sample size was calculated to be 35 cases. Data were analyzed using SPSS version 21 and STATA 11. Qualitative and quantitative variables were presented with frequency and percentage, and mean ± standard deviation, respectively. For evaluating the screening performance characteristics of AWU and LWE in prediction of need for laparotomy sensitivity, specificity, positive predictive value (PPV), negative predictive value (NPV), positive likelihood ratio (PLR), and negative likelihood ratio (NLR) with 95% CI were calculated. The area under the receiver operating characteristic (ROC) curve of the two tests was compared. Need for laparotomy based on final decision of in charge surgeon was considered as the reference test. P-values less than 0.05 were assumed significant results. 

## Results

***Baseline characteristics***

50 cases with the mean age of 28.44 ± 7.14 years (18 – 47) were included (80% male). Table 1 shows the baseline characteristics of studied patients. 46% of cases were in 25 – 35 years age group, 61.7% single, 72% employee, and 82% with knife. 3 (6%) cases were discharged against medical advice and were omitted from final analysis. 

***Screening characteristics***

Based on AWU and LWE 14 (28%) and 17 (34%) cases were detected as peritoneal penetration and needed laparotomy. According to the final decision of surgery service based on all clinical and imaging findings, 17 (34%) cases underwent laparotomy during the hospitalization period. 

**Figure 2:**
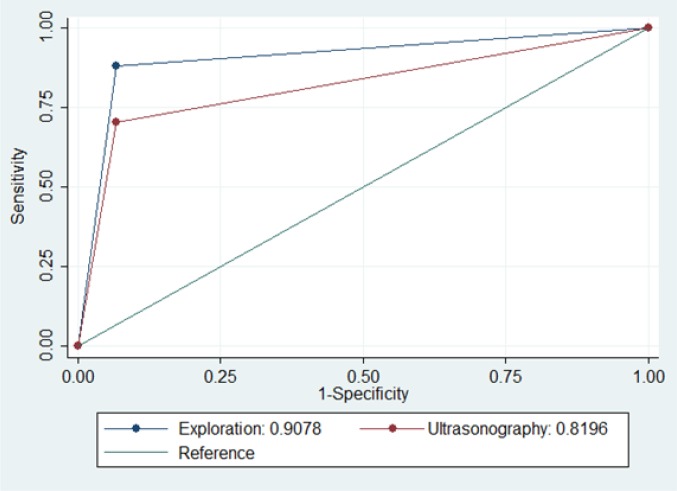
Area under the receiver operating characteristic (ROC) curve of abdominal wall ultrasonography and local wound exploration in prediction of need for laparotomy following anterior abdominal stab wound (p = 0.0641).

Table 2 and figure 2 summarize the screening performance characteristics of AWU and LWE in prediction of need for laparotomy following anterior abdominal stab wound. The area under the ROC curve of AWU and LWE were 81.96 (95% CI: 69.91 – 94.01) and 90.78 (95% CI: 81.67 – 99.89), respectively (p = 0.0641).

## Discussion

Based on the findings of the present study, AWU and LWE have the same specificity (93.3%) but different sensitivities (70.58% versus 88.23%) in screening of anterior abdominal stab wound patients in need of laparotomy. The overall accuracy of LWE in this regard is slightly higher (91.48% versus 85.1%) without statistical significance. 

**Table 1:** Baseline characteristics of studied patients (n = 50)

**Variable**	**Number (%)**
**Age (year)**	
18 -24.9	12 (24)
25- 34.9	23 (46)
≥ 35	15 (30)
**Sex**	
Male	40 (80)
Female	10 (20)
**Marital status**	
Married	18 (38.3)
Single	29 (61.7)
**Trauma mechanism**	
Knife	41 (82)
Other	9 (18)
**Employment**	
Employed	36 (72)
Non-employed	14 (28)
**Vital Sign**	
Systolic blood pressure (mmHg)	117.10 ± 10.79
Diastolic blood pressure (mmHg)	71.30 ± 9.36
Heart rate (/minute)	87.62 ± 6.97
Respiratory rate (/minute)	19.20 ± 1.84
Oxygen saturation (%)	96.52 ± 1.50

Data are presented as mean ± standard deviation or frequency and percentage.

**Table 2:** Screening performance characteristics of abdominal wall ultrasonography (AWU) and local wound exploration (LWE) in prediction of need for laparotomy following anterior abdominal stab wound (n = 47

**Characteristics**	**AWU**	**LWE**
True positive	12 (25.5)	15 (31.9)
True negative	28 (59.6)	28 (59.6)
False positive	2 (4.2)	2 (4.2)
False negative	5 (10.6)	2 (4.2)
Sensitivity	70.58 (44.04 – 88.62)	88.23 (62.25 – 97.93)
Specificity	93.33 (76.49 – 98.83)	93.33 (76.49 – 98.83)
Positive predictive value	85.71 (56.15 – 97.48)	88.23 (62.25 – 97.93)
Negative predictive value	84.84 (67.33 – 94.28)	93.33 (76.49 – 98.83)
Positive likelihood ratio	6.00 (1.63 – 22.03)	7.50 (2.01 – 27.88)
Negative likelihood ratio	0.17 (0.07 – 0.40)	0.07 (0.01 – 0.27)

Data are presented with 95% confidence interval.

Rapid assessment and decision making plays a main role in improving the outcome of severely injured trauma patients (8-11). Although using LWE could be helpful in eliminating hospitalization of more than 30% of patients with anterior abdominal stab wound (2), it is invasive, uncomfortable for the patient, and difficult in obese and uncooperative patients as well as those with thick abdominal musculature. Finding noninvasive alternatives for LWE is an interesting area in management of these patients in emergency and surgery departments.

Ultrasonography is known as an available diagnostic measure for focused assessment of blunt and penetrating trauma patients in emergency department (12-17). Omari et al. showed that ultrasonography is a good guide for selecting patients in need for laparotomy following penetrating abdominal trauma (1). Murphy et al. reported that tract ultrasonography in patients with anterior abdominal penetrating trauma had 59% sensitivity and 100% specificity (18). Soffer et al. reported that sonography had 48% sensitivity and 98% specificity in diagnosis of intra-abdominal lesions and Fray et al. reported 100% positive and negative predictive value of ultrasonography in this regard (19, 20). Ku et al. presented a 76 year-old stab wound case with negative abdominal CT scan findings, which underwent laparoscopy based on positive tract ultrasonography finding regarding peritoneal impairment (21). 

In our study, need for laparotomy was confirmed in 85.7% of patients with positive AWU and 88.2 % of positive LWE results. There were 5 (10.6%) cases with false negative ultrasonography reports and 2 (4.2%) cases with false negative exploration reports. Although the difference in total accuracy of the two models is not statistically significant, accuracy of exploration is in excellent range and ultrasonography in good range.

As we know, ultrasonography is very operator dependent and this slight inferiority of ultrasonography could be eliminated by more practice. 

It seems that tract ultrasonography as a bedside, noninvasive, non-expensive, available, and safe diagnostic approach could be considered for screening of penetrating abdominal trauma patients in need of laparotomy. 

***Limitation***

Among the limitations of the present study is not evaluating the cases with multiple penetrating traumas and those with trauma of other parts of the abdominal area. In addition, in most cases trauma was caused by knife, which affects the diversity of trauma type and might limit the generalizability of the results for cases such as wounds caused by bullets. Patient selection for laparotomy was done by surgeons who were aware of the results of LWE, which might have led to some type of selection bias.

## Conclusion

Based on the findings of the present study, AWU and LWE have the same specificity (93.3%) but different sensitivities (70.58% versus 88.23%) in screening of anterior abdominal stab wound patients in need of laparotomy. The overall accuracy of LWE is slightly higher (91.48% versus 85.1%) without statistical significance. 
